# Earable POCER: Development of a Point-of-Care Ear Sensor for Respiratory Rate Measurement

**DOI:** 10.3390/s18093020

**Published:** 2018-09-10

**Authors:** Kazuhiro Taniguchi, Atsushi Nishikawa

**Affiliations:** 1 Graduate School of Information Sciences, Hiroshima City University, 3-4-1 Ozukahigashi, Asaminami-ku, Hiroshima 731-3194, Japan; 2 Faculty of Textile Science and Technology, Shinshu University, 3-15-1 Tokida, Ueda, Nagano 386-8567, Japan; nishikawa@shinshu-u.ac.jp; 3 Division of Biological and Medical Fibers, Institute for Fiber Engineering (IFES), Interdisciplinary Cluster for Cutting Edge Research (ICCER), Shinshu University, 3-15-1 Tokida, Ueda, Nagano 386-8567, Japan

**Keywords:** ear sensor, optical measurement, respiratory rate, point-of-care testing

## Abstract

We have carried out research and development on an earphone-type respiratory rate measuring device, earable POCER. The name earable POCER is a combination of “earable”, which is a word coined from “wearable” and “ear”, and “POCER”, which is an acronym for “point-of-care ear sensor for respiratory rate measurement”. The earable POCER calculates respiratory frequency, based on the measurement values over one minute, through the simple attachment of an ear sensor to one ear of the measured subject and displays these on a tablet terminal. The earable POCER irradiates infrared light using a light-emitting diode (LED) loaded on an ear sensor to the epidermis within the ear canal and, by receiving that reflected light with a phototransistor, it measures movement of the ear canal based on respiration. In an evaluation experiment, eight healthy subjects first breathed through the nose 12 times per minute, then 16 times per minute, and finally 20 times per minute, in accordance with the flashing of a timing instruction LED. The results of these evaluation tests showed that the accuracy of the respiratory frequency was 100% for nose breathing 12 times per minute, 93.8% at 16 times, and 93.8% at 20 times.

## 1. Introduction

Respiration is one of the most important activities for maintaining life. The respiratory rate is used to grasp the state of one’s health in the fields of medicine, nursing, and care. The respiratory rate is counted as one breath comprising inhaling and exhaling one time each. The mean respiratory rate in a healthy adult at rest is approximately 12–20 per minute [1]. The respiratory rate is normally measured when both the body and mind are in a calm state. Respirations are measured visually or by using a stethoscope or electro-cardiogram. At the research stage, there has been a method of estimating respiration from breathing sounds measured with a microphone attached to the chest [2], a method of estimating respiration from pulse sensor values [3,4], methods of measuring respiration from chest movements obtained using camera images [5] or laser range finders [6], a method of measuring respiration based on the electrical impedance in the arm [7], and methods of estimating respiration based on measurement values from flow sensors attached to the mouth [8,9,10,11,12]. These are all beneficial research methods.

The concept of point-of-care testing (POCT) was introduced, mainly in Western countries, in the 1980s; since the second half of the 1990s, we have seen the rapid spread of POCT measuring devices [13]. When considering respiratory rate measurement equipment suited for POCT, it is important not only that the measurements are accurate but, from the initial stages of basic design, that development proceed so that “equipment be lightweight, low-cost, simple to operate, and capable of rapidly starting respiration measurement.” Some of the respiratory measurement devices [2,3,4,5,6,7,8,9,10,11,12] described above can be adapted for POCT, but were not originally studied and developed with the aim of being adapted for POCT. Aiming to develop a respiratory measurement device adapted for POCT, we have studied and developed an earphone-type respiratory measurement device (hereafter, referred to as an “earable POCER”). The name earable POCER is a combination of “earable”, which is a word coined from “wearable” and “ear”, and “POCER”, which is an acronym for “point-of-care ear sensor for respiratory rate measurement”. The earable POCER, automatically measure respiration frequency through the attachment of an earphone-sensor (hereafter referred to as an “ear sensor”) to the right ear or left ear of the measured subject and displays the results on a tablet terminal. Thus far, using an ear sensor, we have been successful in researching and developing devices for measuring meal times, a measuring device for the number of chewing times, and hands-free interfaces based on results from measuring tongue activity [14,15,16,17,18,19,20]. For this sensor technology for the earable POCER, we applied the ear sensor technology that we developed previously. The ear sensor used in the earable POCER is a photo sensor which, in comparison to methods of measurement that utilize a microphone [21], is strong against environmental noise. Furthermore, when compared to an electrocardiogram, it can measure respiratory rate more rapidly and simpler with lower cost. Compared to the method utilizing a flow sensor, it does not obstruct the mouth or nose, so there is less of a burden on the patient. The earable POCER enables automatic measurement by simply attaching the ear sensor to one ear of the subject to be measured, so there is no need for the measurer to be continuously located in the vicinity of the measured subject, as in the case of visual measurement or measurement utilizing a stethoscope. Research in which the sensor is inserted in the ear, such as the earable POCER, and the respiratory rate is measured and estimated including those where an earphone-type microphone is attached to the ear to measure the respiratory rate [21] and those where the respiratory rate is estimated from photoplethysmography (PPG) measured with the earphone-type sensor [22,23]. Compared to the former, the measurement accuracy of the earable POCER is not affected by environmental noise, whereas the latter, while requiring estimation processing, has a much more complex system configuration compared to the earable POCER. In addition, when estimating the respiratory rate using PPG, it is difficult to distinguish whether patients are breathing by themselves without relying on the artificial respirator or not. The earable POCER can be used to monitor the respiratory functions of patients because the earable POCER only measures independent breathing.

In this paper, we describe the mechanism of an earable POCER prototype and the evaluation results. Specifically, we describe the method of measurement using the ear sensor, and the processing method of calculating the respiratory frequency from the waveform measured using the ear sensor. We also describe the results of an experiment in which we evaluated eight test subjects using this prototype. The prototype in this paper is realized through the use of both measurement technology and signal processing technology, and making the device wireless or compact or user-friendly displays or operational applications (human machine interface) loaded in the tablet terminal are not covered.

## 2. Materials and Methods

### 2.1. Hardware

**Figure 1:**
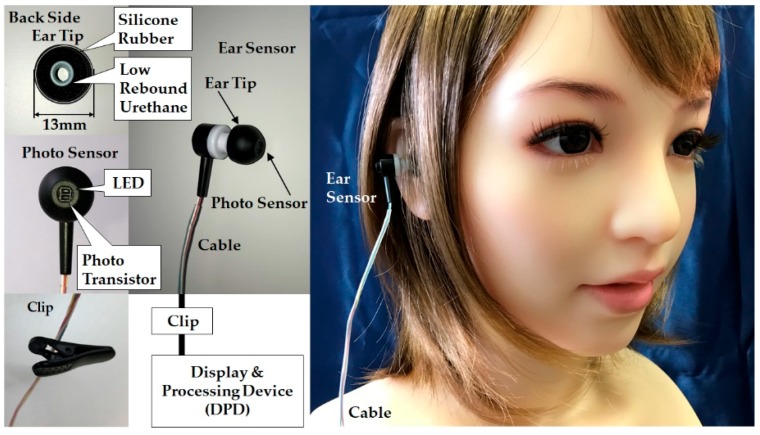
External diagram of the earable point-of-care ear sensor for respiratory rate measurement (POCER). The earable POCER uses an ear sensor attached to one ear. The earable POCER is comprised of one ear sensor for measuring the respiratory rate of the measured subject, a display and processing device (DPD) that calculates and displays the respiratory frequency from the values measured by the ear sensor, and one clip for fixing the cable stretching from the ear sensor to the DPD on the subject’s clothes. A photo sensor is attached to the ear sensor. The surface of the ear tip on the ear sensor is covered with silicone rubber, and the inside is made of low-rebound urethane. Using this silicone rubber and low rebound urethane, the ear sensor fits snugly in the ear hole.

**Figure 2:**
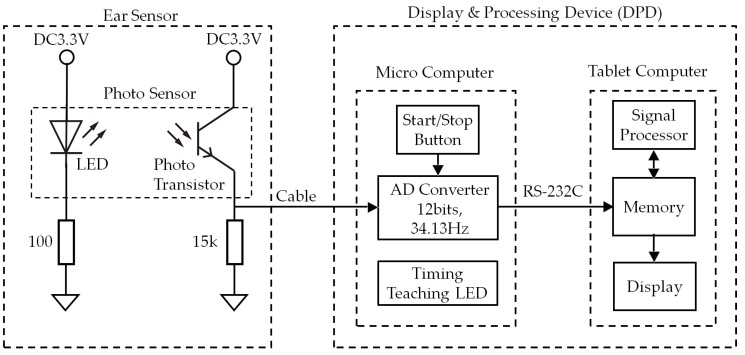
Block diagram of the earable POCER. The photo sensor is equipped with one light-emitting diode (LED) with a wavelength of 940 nm and one photo transistor. The DPD is comprised of an AD converter, start/stop button, timing teaching LED, signal processor, memory, and display. The analog signal measured with the ear sensor is converted into a digital signal with a sampling frequency of 34.13 Hz using the AD converter. The measured values, converted into a digital signal, are recorded in the memory. Based on the information recorded in memory, the respiratory frequency is calculated from the signal processor, and the respiratory frequency and frequency waveform are shown on the display. The timing instruction LED has a duty ratio of 50%, and frequencies with the three patterns of 200 mHz, 267 mHz, and 333 mHz. The start/stop button is used for starting and stopping the earable POCER.

An external diagram of the earable POCER is shown in Figure 1, and a block diagram is shown in Figure 2. As seen in Figure 1, the earable POCER uses an ear sensor attached to one ear. The earable POCER is comprised of one ear sensor for measuring the respiratory rate of the measured subject, a display and processing device (DPD) that calculates and displays the respiratory frequency from the values measured by the ear sensor, and one clip for fixing the cable stretching from the ear sensor to the DPD on the subject’s clothes. A photo sensor is attached to the ear sensor. The surface of the ear tip on the ear sensor is covered with silicone rubber, and the inside is made of low rebound urethane. Using this silicone rubber and low rebound urethane, the ear sensor fits snugly in the ear hole.

As shown in Figure 2, the photo sensor is equipped with one light-emitting diode (LED) with a wavelength of 940 nm and one photo transistor. The DPD is comprised of an analog–digital (AD) converter, start/stop button, timing teaching LED, signal processor, memory, and display.

**Figure 3:**
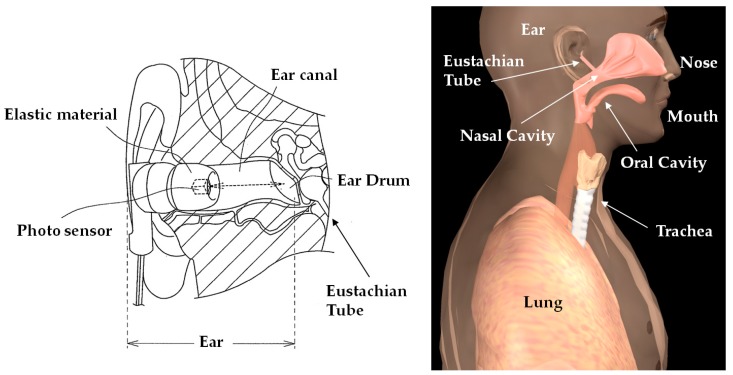
Measurement principles of the ear sensor. The ear sensor has a compact photo sensor attached. The photo sensor irradiates infrared light using an LED to the epidermis of the ear canal, and by receiving the reflected light with a phototransistor, it can measure form changes in the ear canal. The ear canal and a nasal cavity are connected by the eustachian tube. The ear canal and nasal cavity are also connected by the levator veli palatini muscle and tensor veli palatini muscle, which are anatomically close to each other. Based on this, when one breathes, the shape of the ear canal changes. Additionally, as one breathes through the mouth, because the action of opening and closing the mouth results from extension and contraction of the temporalis muscle, this extension and contraction of the temporalis muscle changes the shape of the adjacent ear canal. Usage of the ear sensor enables optical and non-invasive measurement of the aforementioned changes in the shape of the ear canal that occur due to breathing.

Figure 3 shows the measurement principles of the ear sensor. The ear sensor has a compact photo sensor QRE1113 (Fairchild Semiconductor International Inc., Sunnyvale, CA, USA) attached. The photo sensor irradiates infrared light using an LED to the epidermis of the ear canal, and by receiving the reflected light with a phototransistor, it can measure form changes in the ear canal. The ear canal and a nasal cavity are connected by the eustachian tube. The ear canal and nasal cavity are also connected by the levator veli palatini muscle and tensor veli palatini muscle, which are anatomically close to each other. Based on this, when one breathes, the shape of the ear canal changes. Additionally, as one breathes through the mouth, because the action of opening and closing the mouth results from extension and contraction of the temporalis muscle, this extension and contraction of the temporalis muscle changes the shape of the adjacent ear canal. Usage of the ear sensor enables optical and non-invasive measurement of the aforementioned changes in the shape of the ear canal that occur due to breathing.

As shown in Figure 2, the analog signal measured with the ear sensor is converted into a digital signal with a sampling frequency of 34.13 (=2048 ÷ 60) Hz using the AD converter. The measured values, converted into a digital signal, are recorded in the memory. Based on the information recorded in the memory, the respiratory frequency is calculated from the signal processor, and the respiratory frequency and frequency waveform are shown on the display. The algorithm used to calculate the respiratory frequency using the signal processor is discussed in Section 2.2. In the evaluation test discussed in Section 3, the timing instruction LED in Figure 2 directs the subject when to breathe and consists of one blue LED. This timing instruction LED has a duty ratio of 50%, and frequencies with the three patterns of 200 mHz, 267 mHz, and 333 mHz. The start/stop button is used for starting and stopping the earable POCER.

The DPD in this paper is used to evaluate the measurement method using the ear sensor and the signal-processing algorithm, and is a simple prototype using a microcomputer and a tablet terminal. The DPD in this paper is realized using a mbed LPC1768 microcomputer (switch science Inc., Tokyo, Japan) that combines the three functions of AD converter, timing instruction LED, and signal processor, with software developed in-house (C language), and the start/stop button uses a tactile switch. This is realized using a tablet terminal surface Pro3 (Microsoft Corp., Redmond, WA, USA), combining the signal processor, memory, and display, and the spreadsheet software Microsoft Excel 2013 (Microsoft Corp., Redmond, WA, USA). A microcomputer and the tablet terminal use a wired USB connection, and communicate using the RS-232C standard protocol. CoolTermWin Version 1.4.7 (Freeware) is used for the communication software.

### 2.2. Algorithm

We shall now explain the respiratory frequency measurement algorithm using the signal processor. This algorithm calculates the respiratory frequency through the utilization of a median filter, band-pass filter with a passband between 189 mHz and 504 mHz, and fast Fourier transform (FFT). First, the ear sensor measurement value is converted to a digital signal using the AD converter, with a sampling frequency of 34.13 Hz, and a resolution of 12 bits. By setting the sampling frequency to 34.13 Hz, data can be displayed at a maximum of 34.13 frames per second. The AD converted value is sent to both memory and the signal processor. At this time, the most recently measured data is set to *v*_1_, the data measured 29.30 $\times 10^{-3}$ (= 60 ÷ 2048) × 1 s before is set to *v*_2_, and the data measured 29.30 × 2 ms before is set to *v*_3_. Using Equation (1) simultaneously with the *v*_1_ measurement, the median value *e* of the data for these 3 periods is calculated with the signal processor and sent to the memory.

(1) $$\;e=\mathrm{Me}\left(v_{1},v_{2},v_{3}\right)\;$$

Here, Me is the function for obtaining the median value of the three figures. By taking the median value, noise components that occur unexpectedly can be excluded, i.e., here, we carry out median filter processing.

In memory, the most recent 80 items (2.4 s = 60 ÷ 2048 × 80) of *e* required for the band-pass filter carried out from Equations (2) to (4) are recorded in a first in, first out (FIFO) system. Therefore, the measurement values recorded in the memory are the 80 median values *e_i_* (*i* = 1, 2, 3, …, 80) obtained from the 82 data items measured using the ear sensor every 29.30 ms. The most recent data is set to *e*_1_, the data calculated 29.30 × 1 ms before is set to *e*_2_, the data calculated 29.30 × 2 ms before is set to *e*_3_…, and the data calculated 29.30 × 79 ms before is set to *e*_80_.

Next, based on Equation (2), *m*_30_ is the mean value obtained from the 30 data items from *e*_51_ to *e*_80_. This calculation smooths out the fluctuations in the values from *e*_51_ to *e*_80_.

(2) $$\;m_{30}=\frac{1}{30}\overset{80}{\underset{j=51}{\sum }}e_{j}\;$$

Additionally, according to Equation (3), *m*_80_ is the mean value obtained from the 80 data items from *e*_1_ to *e*_80_. This calculation smooths out the fluctuations in the values from *e*_1_ to *e*_80_.

(3) $$\;m_{80}=\frac{1}{80}\overset{80}{\underset{j=1}{\sum }}e_{j}\;$$

Furthermore, based on Equation (4), *r* is the difference in values from *m*_30_ to *m*_80_, and *r* is sent to the memory. *r* is stored in the memory, with up to 2048 items using the FIFO system, and these are set to *r_i_* (*i* = 1, 2, 3, …, 2048).

(4) $$\;r=m_{30}-m_{80}\;$$

The processing from Equations (1) to (4) is carried out every 29.30 ms when data is measured and sent. In other words, Equations (2) and (3) obtain the moving average of the value acquired in Equation (1). The moving average plays the role of a lowpass filter. Based on the moving average, the lowpass filter cut-off frequency *f*_c_ is obtained in Equation (5).

(5) $$\;f_{c}=\frac{0.443}{\sqrt{n^{2}-1}}f_{s}\;$$

Equation (5) shows the frequency *f*_c_ in which the gain *G* of frequency characteristic of the moving averages, given by Equation (6), is $1/\sqrt{2}$. *f*_s_ is the sampling frequency, which is 34.13 Hz. Δt is the sampling frequency, and the value is 1/*f*_s_.

(6) $$\;G=\frac{1}{2\pi f_{c}n\Delta t}\sqrt{2\left(1-\cos2\pi f_{c}n\Delta t\right)}\;$$

In Equation (2), *n* is 30, whereas in Equation (3), *n* is 80. The cut-off frequency *f*_c_ is 504 mHz in Equation (2), and 189 mHz in Equation (3). Equation (4) is the band-pass filter processing in the passband from 189 mHz to 504 mHz. The moving average does not have an ideal lowpass filter function, and so some frequency elements other than those in the passband may pass, even if they are attenuated.

FFT analysis is performed using *r_i_* data. However, FFT analysis when the signal length is 2048 has a high calculative burden. Therefore, by extracting one of every 16 items from the 2048 items of *r_i_*, and thus thinning it down to 128 items (*r*_1_, *r*_128_, *r*_256_, …, *r*_2048_), the data length and thus the calculation load can be reduced. Additionally, to reduce the direct current (DC) components from *r_i_*, we obtained the mean of *r*_1_, *r*_128_, *r*_256_, …, *r*_2048_, and set that mean to $\bar{r}$, and for FFT analysis, used *r*_1_ − $\bar{r}$, r_128_ − $\bar{r}$, *r*_256_ − $\bar{r}$, …, *r*_2048_ − $\bar{r}$ with that mean subtracted.

Because the sampling frequency is 2.13 (=2048 ÷ 16 ÷ 60) Hz, and the signal length is 128 (= 2048 ÷ 16), it is possible to obtain the frequency spectrum in 1/60 Hz increments. In other words, the respiratory rate can be obtained as a natural number per increment. The maximum frequency from the calculated frequency spectrum multiplied by 60 is the respiratory rate. With the horizontal axis of the display device, every time *r_i_* is calculated, the time (29.30 ms intervals), and the vertical axis is the most recent value of *r_i_*, with this becoming the respiratory waveform. This respiratory waveform can be depicted using a maximum of approximately 34.13 items of data per second.

For the purposes of analysis after the experiment, the 2129 items of measured values *v_i_* (*i* = 1, 2, 3, …, 2129) obtained in experiment 3.2 are stored in the memory. Here, there are over 2048 items of FFT data, an additional 79 items of data are required for the band-pass filter, and a further additional 2 items are required for the median filter, making a total of 2129 items of data.

## 3. Evaluation Experiments

### 3.1. Subjects

The subjects consisted of eight healthy people (males and females between the ages of 22 and 62 years old, mean age 38.3 years old), and these were referred to, respectively, as A, B, C, D, E, F, G, and H. The subjects attached the ear sensors to their ear; it was confirmed that these were neither too large nor too small, and those who complained of experiencing ear pain or fatigue, as well as those who were undergoing treatment of some sort, were excluded. Additionally, those experiencing no respiratory problems were selected as subjects. Those people who experienced discomfort when attaching the ear sensor were excluded.

The study was conducted in accordance with the Declaration of Helsinki, and received the approval of the “Ethics Review Procedures concerning research with human subjects at Shinshu University (project identification code: 182)”. The subjects received a full explanation in advance and provided their consent to participate in the study. Additionally, in regard to matters of hygiene for all experiments, the ear sensors were cleaned with a cleaning brush before and after use, and these were disinfected using ethanol disinfectant.

### 3.2. Earable Point-of-Care Ear Sensor for Respiratory Rate Measurement (POCER) Evaluation Experiments

To evaluate the measurement method from Section 2.1 and the respiratory frequency measurement algorithm from Section 2.2, we performed the following experiment. The earable POCER (ear sensor to right ear of subject, clipped to clothes) was attached and the subjects were asked to breathe through their nose 6 times for approximately 1 min each time (sampling frequency 29.30 ms, with 2129 total samples), with the first and second set performed by breathing through the nose in accordance with the 200 mHz flashing of the timing instruction LED at a pace of 12 times per minute (this experiment was referred to as “EX200mHz12rpm”). In the same way, the third set and fourth set were performed by breathing through the nose in accordance with the 267 mHz flashing of the timing instruction LED at a pace of 16 times per minute (this experiment was referred to as “EX267mHz16rpm”). For the fifth and sixth sets, they breathed through the nose in accordance with the 333 mHz flashing of the timing instruction LED at a pace of 20 times per minute (this experiment was referred to as EX333mHz20rpm”). In the above experiment, the subject breathed in when the timing instruction LED lit up and breathed out when the light went out. In this experiment, the frequency of the timing instruction LED is the true value of the respiratory frequency.

In this experiment, each measurement was started by pressing the start/stop button in Figure 2, and automatically ended 62.37 s later (after 2129 items of data had been collected). The measurement could be ended at any time during the course of the measurement by pressing the start/stop button.

## 4. Results

Table 1 shows the results of the evaluation experiments using the eight subjects in Section 3. Item represents the content of experiment, and EX200mHz12rpm, EX267mHz16rpm, and EX333mHz20rpm are the three types. The next column, FFT [mHz], indicates the frequency at which the FFT power spectrum was maximum in the Item experiment. This frequency corresponds to the calculated value of the respiratory frequency (calculated by the earable POCER). Finally, as a reference value, we obtained the wave number (number of peaks) through zero-crossing detection from the values used in FFT analysis. This is the reference value for the respiratory rate. This zero-crossing detection considers the waveform peaks changing “from negative to positive, and then positive to negative”, as one time each time there is a progression in time, and it counts the total number of peaks during one minute. In this experiment, each of the eight subjects performed the 200 mHz, 267 mHz, and 333 mHz experiments twice, and so there were 16 readings of data for EX200mHz12rpm, EX267mHz16rpm, and EX333mHz20rpm, respectively. The average value was calculated by averaging the data of 16 times. Similarly, “accuracy”was also obtained by dividing the number of people for whom the respiratory frequency calculated value was equivalent to a true value (frequency of the timing instruction LED) by 16. The closer the accuracy is to 1, the more accurately the earable POCER is considered to have measured the respiratory frequency of the subjects.

As shown in Table 1, the FFT results for subjects A, C, D, E, F and G are equivalent to true values, and the respiratory frequencies were measured correctly. For subject B, for both of EX200mHz12rpm and EX333mHz20rpm, as well as the second time for EX267mHz16rpm, the respiratory frequency was measured accurately; however, the value for the first time for EX267mHz16rpm was 17 mHz, which was different from the true value. In the first FTT result for EX267mHz16rpm, the second largest power spectrum frequency was equivalent to the true value at 267 mHz. For subject H, during both times for EX200mHz12rpm and EX267mHz16rpm, as well as the first time for EX333mHz20rpm, the respiratory frequency was measured accurately, but the value for the second EX333mHz20rpm was 17 mHz, which was different from the true value. However, for the second FTT result for Ex333mHz20rpm, the second largest power spectrum frequency was equivalent to the true value at 333 Hz. The mean values obtained from the 16 (=8 subjects × 2 times) respiratory frequencies using FFT were 200.0 mHz for EX200mHz12rpm, 251.4 mHz for EX267mHz16rpm, and 313.3 mHz for EX333mHz20rpm. Additionally, the respiratory frequency accuracy was 1.000 for EX200mHz12rpm, 0.938 for EX267mHz16rpm, and 0.938 for EX333mHz20rpm. Next, the mean values for the respiratory rate for the 16 data obtained from the number of peaks was 12.1 for EX200mHz12rpm, 15.4 for EX267mHz16rpm, and 19.2 for EX333mHz20rpm. Furthermore, the respiratory rate accuracy was 1.000 for EX200mHz12rpm, 0.750 for EX267mHz16rpm, and 0.500 for EX333mHz20rpm. In case a margin of error of ±1 time was permitted, the respiratory rate accuracy was 0.875 (=14/16) for EX267mHz16rpm and 0.813 (=13/16) for EX333mHz20rpm.

**Figure 4:**
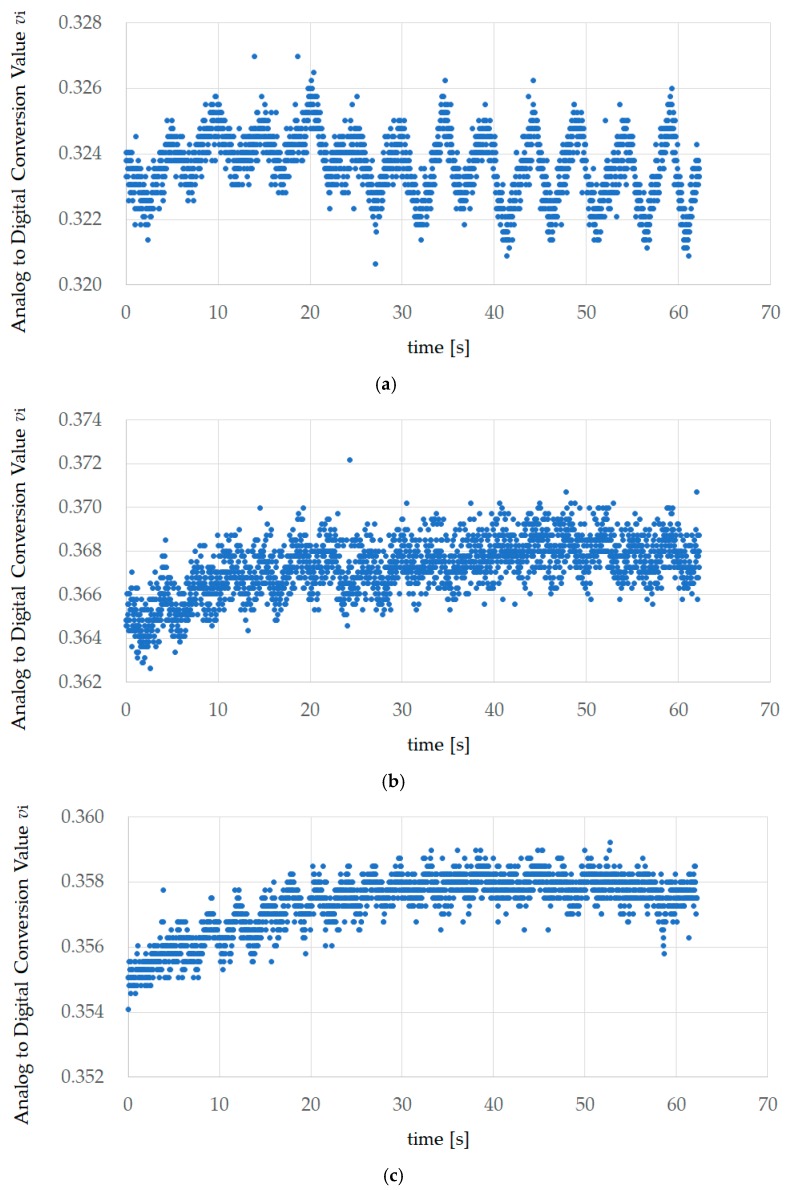
Experiment results of EX200mHz12rpm, EX267mHz16rpm, and EX333mHz20rpm for subject A. This value is the analog–digital (AD) converted value *v*_i_ measured using the sensor. A.D. conversion occurred with a sampling frequency of 34.13 Hz and a resolution of 12 bits, and the number of data items was 2129. The measurement time was approximately 62.37 s. (**a**) The first experiment results of 200 mHz (EX200mHz12rpm); (**b**) the first experiment results of 267 mHz (EX267mHz16rpm); (**c**) the first experiment results of 333 mHz (EX333mHz20rpm).

Figure 4 shows the first experiment results of EX200mHz12rpm, EX267mHz16rpm, and EX333mHz20rpm for subject A. This value is the AD converted value *v_i_* measured using the sensor. AD conversion occurred with a sampling frequency of 34.13 Hz and a resolution of 12 bits, and the number of data items was 2129. The measurement time was approximately 62.37 s.

**Figure 5:**
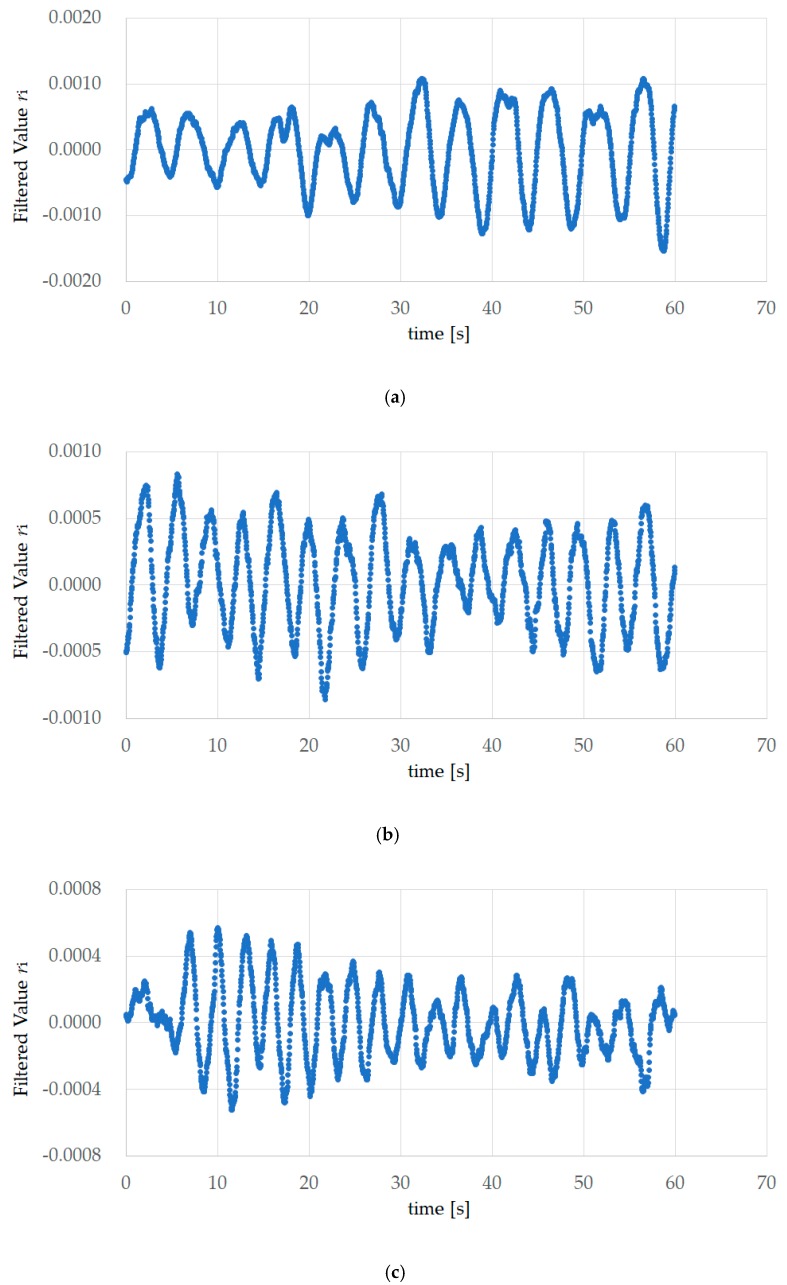
FFT processing data obtained from experiment results of EX200mHz12rpm, EX267mHz16rpm, and EX333mHz20rpm for subject A. Median filter processing, band-pass filter processing with a passband from 189 mHz to 504 mHz and thinning to 1/16 for the 2048 items was performed on *v*_i_ in Figure 4, and processing was performed so that the mean value of the 128 data items was 0, creating the data for one minute. (**a**) The first experiment results of 200 mHz (EX200mHz12rpm); (**b**) the first experiment results of 267 mHz (EX267mHz16rpm); (**c**) the first experiment results of 333 mHz (EX333mHz20rpm).

Figure 5 shows the FFT processing data obtained from the results of the first EX200mHz12rpm, EX267mHz16rpm, and EX333mHz20rpm experiments for subject A. Median filter processing, band-pass filter processing with a passband from 189 mHz to 504 mHz, and thinning to 1/16 for the 2048 items was performed on *v_i_* in Figure 4, and processing was performed so that the mean value of the 128 data items was 0, creating the data for one minute. The total number of peaks in Table 1 corresponds to the wave number obtained using zero-crossing detection, and represents the reference value for the respiratory rate. For example, the wave numbers in Figure 5b is 16, and the reference value for the respiratory rate is 16 times.

**Figure 6:**
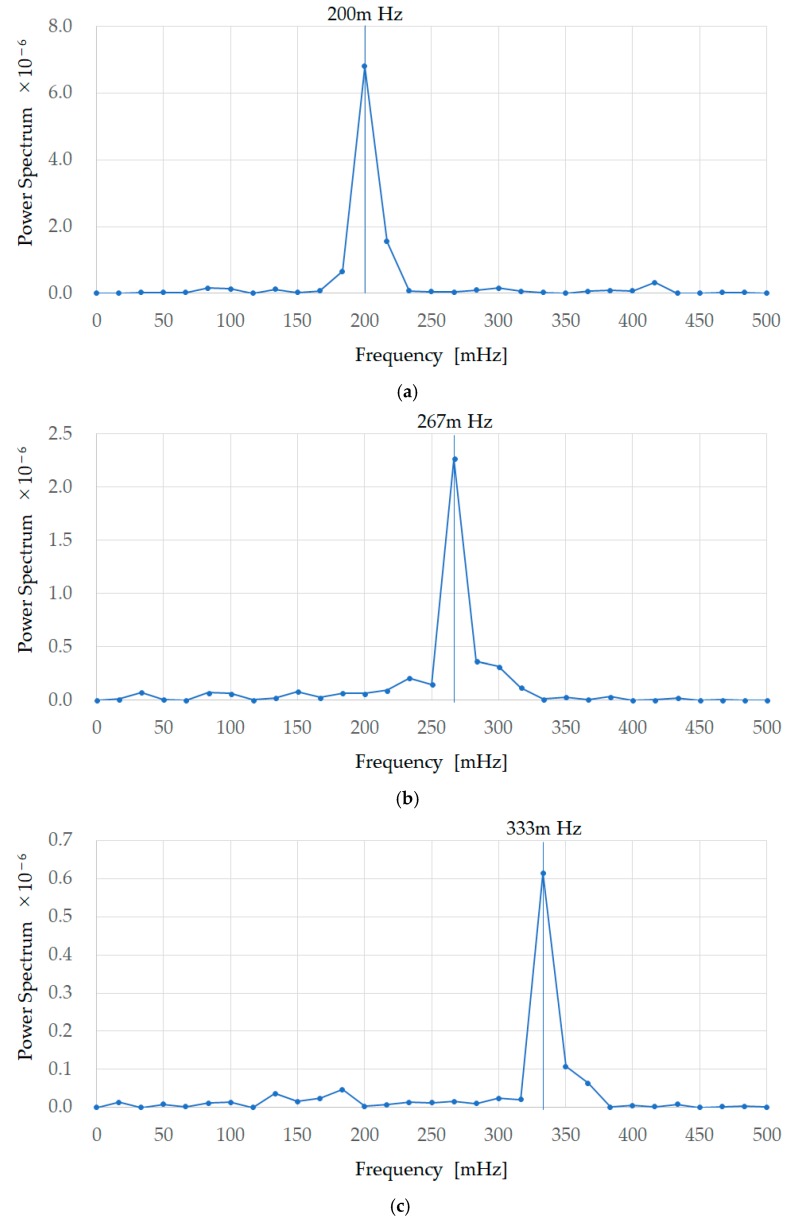
FFT processing results for the experiment results of EX200mHz12rpm, EX267mHz16rpm, and EX333mHz20rpm for subject A. These are the results of performing FFT with a data length of 128 on the data in Figure 5, and show that the true values (timing instruction LED frequencies) and earable POCER calculated values (frequencies at which the power spectrum is maximum) match. (**a**) The first experiment results of 200 mHz (EX200mHz12rpm); (**b**) the first experiment results of 267 mHz (EX267mHz16rpm); (**c**) the first experiment results of 333 mHz (EX333mHz20rpm).

Figure 6 shows the FFT processing results of the first EX200mHz12rpm, EX267mHz16rpm, and EX333mHz20rpm experiments for subject A. These are the results of performing FFT with a data length of 128 on the data in Figure 5. The frequency at which the power spectrum is at its maximum is 200 mHz, 267 mHz, and333mHz respectively, and a summary of the frequencies at which the power spectrum was maximum is shown, similarly, for all subjects as FFT in Table 1. Figure 6 shows that the true values (timing instruction LED frequencies) and earable POCER calculated values (frequencies at which the power spectrum is maximum) match.

## 5. Discussion

As the accuracy in Table 1 is 1.000 for EX200mHz12rpm, 0.938 for EX267mHz16rpm, and 0.938 for EX333mHz20rpm, from this we can see that the earable POCER has the ability to correctly measure and calculate the respiratory frequency for the subjects. However, in the case of EX267mHz16rpm, the measurement results for the first time for subject B were wrong one time. Additionally, in EX333mHz20rpm, the measurement results for subject H were wrong one time—the second time. If the earable POCER is used in the medical field, the output of incorrect values, as shown in these two cases, would be problematic. To resolve this problem, a self-check function should be added to the earable POCER. As a self-check method, for example, the value of multiplying the FFT calculated value by 60 can be compared to the total number of peaks value, and if there is a gap of 30% or more, it would be considered a value with low reliability, and would be measured and calculated again. Alternatively, “this is a value of low reliability” could be displayed along with the respiratory frequency measurement results, thus communicating to the user that the calculation results may be wrong.

When comparing the FFT calculation values and the total number of peaks values, experimental results with a gap of 30% or more occurred during the first time for EX267mHz16rpm for subject B and the second time for EX333mHz20rpm for subject H; in both of these cases, the respiratory frequency value differed from the true value, indicating that the method described above may detect incorrect values. We have proposed 30% as the criteria. This value was obtained as the value at which, while the FFT values and true values were the same, the total number of peaks value was furthest from the true value. More specifically, the FFT (respiratory frequency) values for the second time for EX333mHz20rpm for subject B and the second time for EX333mHz20rpm for subject E were the same as the true values, but the total number of peaks differed by three times the true value of 20 times. By dividing this difference of three times by the true value of 20 times, and then, as a safety rate for the value, multiplying this by two, we obtained the criteria of 0.3 (30%). This criteria value is a provisional value and further investigation is required.

As the total number of peaks was counted using zero-crossing detection in Section 4, non-zero crossed peaks were not counted. In other words, even when there was a peak, the figures forming that peak were all positive values, and where there were negative values, they were not counted. In the experiment results for the second time for EX333mHz20rpm for subject B and the second time for EX333mHz20rpm for subject E, there were several non-zero crossed peaks, and when these were added, subject B had 19 times and subject E 20 times, approaching the true values.

During this evaluation experiment, some of the test subjects were overly conscious of matching their breathing to the flashing of the timing instruction LED and breathed more strongly than they normally would. It is unclear what effect this had on the experiment results, but the experimental method may be re-examined as necessary. Additionally, in the evaluation test this was a nose breathing experiment, but we also plan experiments using mouth breathing, and combinations of nose breathing and mouth breathing.

For this prototype, we designed a band-pass filter so that it could accurately measure when the respiratory rate is kept within a range of 12 to 20. In activities such as meditation or yoga, extremely slow abdominal breathing may take place, and it is possible that the respiratory rate will fall below 12 times. Moving forward, we would like to review the filter processing and investigate other methods to broaden the range of frequencies that can be measured. Furthermore, we are currently performing FFT based on the measurement results during one minute, and calculating the respiratory frequency, but we shall also work to shorten the examination time, through the cooperative use of methods that calculate the frequency cycle from peak to peak of the waveform after filter processing.

As described in Section 1, the prototype in this paper is realized using both measurement technology and signal processing technology but does not cover making the device wireless or compact, or user-friendly displays or operational applications (human interface) loaded in the tablet terminal. Based on the goal that the “equipment be lightweight, low-cost, simple to operate, and capable of rapidly starting respiration measurements”, which is important when developing a POCT device, we would like to keep improving the finished areas and developing the unfinished areas. We would also like to carefully consider the issues one by one when actually operating the earable POCER in the medical field, and perform experiments under various conceivable conditions, to improve the earable POCER and make it more practical. More specifically, during the next step we plan to study and evaluate patient compliance factors with an internal physician. Moving forward, we would like to demonstrate the clinical impact/importance, including patient compliance, of the earable POCER. Additionally, we plan to develop testing protocol(s) for measuring normal/unsupervised breathing rates, and use a statistical method, such as Bland–Altman plots, for validating their device against a gold standard. Additionally, in recent years, there has been research and development (R&D) into wearable sensors that can measure and estimate the respiratory rate from a physical position outside the ear. These include, for example, wrist worn PPGs [24], chest mounted accelerometers [25], and electrocardiogram (ECG) patches [26,27]. We plan to investigate, with the internal physician, which sensor can be leveraged most easily with POCT. Finally, by publishing the study results for the earable POCER and the comparative results with other equipment, we aim to contribute toward the development of POCT.

## 6. Conclusions

We have carried out research and development on an earphone-type respiratory rate measuring device, which we aim to adapt to point-of-care testing (POCT). The earable POCER, simply by attaching an earphone-type sensor (ear sensor) to one ear of the measured subject, calculates respiratory frequency based on the measurement values over one minute, and displays these results on a tablet terminal. 

In an evaluation experiment, we had 8 healthy subjects A to H (males and females from the ages of 21- to 62-years-old, mean age 38.3-years-old), attach the earable POCER and perform six sets of approximately one minute of breathing through the nose. In the first two sets, they breathed through the nose at a pace of 12 times per minute in accordance with the flashing of a timing instruction LED. Similarly, they breathed through their nose at a pace of 16 times in the third and fourth sets, and 20 times in the fifth and six sets. The results of these evaluation tests were that the mean value of respiratory frequency for the eight subjects obtained from the earable POCER was 200.0 mHz for the nose-breathing experiments 12 times per minute (200 mHz), 251.4 mHz for 16 times (267 mHz), and 313.3 mHz for 20 times (333 mHz). Additionally, the accuracy of the respiratory frequency was 100% for nose breathing 12 times per minute, 93.8% for 16 times, and 93.8% for 20 times. During this experiment, none of the subjects felt the sensor to be an obstruction to respiration. Furthermore, none of the subjects experienced fatigue or poor health during the experiment.

Moving forward, we plan to expand the measurement range by improving filter processing and accuracy through the cooperative use of methods other than FFT, such as measuring the frequency between peak-to-peak intervals of the waveform after filter processing, as well as working to shorten the measurement time. Additionally, we would also like to carefully consider issues involved with actually operating the earable POCER in the medical field one by one, and perform experiments under various conceivable conditions, in order to improve the earable POCER and make it more practical.

## Figures and Tables

**Table 1:** Evaluation experiment results for the eight subjects. Item represents the content of experiment. The next column, fast Fourier transform (FFT) (mHz), indicates the frequency at which the FFT power spectrum was maximum in the Item experiment. This frequency corresponds to the calculated value of the respiratory frequency (calculated by the earable POCER). Finally, as a reference value, we obtained the wave number (number of peaks) through zero-crossing detection from the values used in FFT analysis. This is the reference value for the respiratory rate.

Subject	Item	FFT (mHz)		Total Number of Peaks (rpm)	
		First	Second	First	Second
A	EX200mHz12rpm	200	200	12	12
	EX267mHz16rpm	267	267	16	16
	EX333mHz20rpm	333	333	19	20
B	EX200mHz12rpm	200	200	12	12
	EX267mHz16rpm	17	267	10	15
	EX333mHz20rpm	333	333	19	17
C	EX200mHz12rpm	200	200	12	13
	EX267mHz16rpm	267	267	16	16
	EX333mHz20rpm	333	333	19	20
D	EX200mHz12rpm	200	200	12	12
	EX267mHz16rpm	267	267	16	16
	EX333mHz20rpm	333	333	19	19
E	EX200mHz12rpm	200	200	12	12
	EX267mHz16rpm	267	267	16	15
	EX333mHz20rpm	333	333	20	17
F	EX200mHz12rpm	200	200	12	12
	EX267mHz16rpm	267	267	16	16
	EX333mHz20rpm	333	333	20	20
G	EX200mHz12rpm	200	200	12	12
	EX267mHz16rpm	267	267	16	16
	EX333mHz20rpm	333	333	20	20
H	EX200mHz12rpm	200	200	12	12
	EX267mHz16rpm	267	267	14	16
	EX333mHz20rpm	333	17	20	18
Average	EX200mHz12rpm	200.0		12.1	
	EX267mHz16rpm	251.4		15.4	
	EX333mHz20rpm	313.3		19.2	
Accuracy	EX200mHz12rpm	1.000		1.000	
	EX267mHz16rpm	0.938		0.750	
	EX333mHz20rpm	0.938		0.500	
